# UK Experience of an Alternative ATO Dosing Regimen in APL

**DOI:** 10.3389/fonc.2020.594129

**Published:** 2020-11-11

**Authors:** Nigel Russell, Richard Dillon

**Affiliations:** ^1^ Department of Haematology, Guy’s and St Thomas’ Hospitals NHS Trust, London, United Kingdom; ^2^ Cancer Genetics Laboratory, Department of Medical and Molecular Genetics, King’s College, London, United Kingdom

**Keywords:** acute promyelocytic leukaemia (APL), arsenic trioxide, minimal residual disease, gemtuzumab ozagamicin, NCRI AML17

## Abstract

The introduction of all-trans retinoic acid (ATRA), and more recently of arsenic trioxide (ATO) in the treatment of Acute Promyelocytic Leukaemia (APL), has been instrumental in achieving the high cure rates recently reported. For the majority of patients, it is now possible to successfully treat this disease “chemo-free” without the use of cytotoxic chemotherapy as reflected in current clinical guidelines. The Sanz risk score developed by the GIMEMA and PETHEMA groups categorizes patients into three risk groups—low, intermediate, and high and correlates with relapse-free survival (RFS). Low- and intermediate-risk APL are now often considered together as ‘standard-risk’ defined by a white blood cell count (WBC) of less than 10 x 10^9^/L. High-risk APL has a WBC greater than 10 x 10^9^/L. In the UK our approach for patients with standard risk APL is to treat with ATRA and ATO without the use of cytotoxic chemotherapy. This approach is based on results from two large randomized clinical trials. The GIMEMA APL0406 trial showed an overall survival advantage compared to anthracycline-based chemotherapy plus ATRA. The UK NCRI AML17 trial which used an attenuated dose of ATO demonstrated a significant reduction in relapse and improved relapse-free survival. In the UK, the National Institute for Clinical Excellence approved both ATO plus ATRA regimens for re-imbursement for standard risk Acute Promyelocytic Leukaemia (APL). We use the AML17 schedule in standard-risk patients upfront and also in patients with relapsed Acute Promyelocytic Leukaemia (APL) previously treated with chemotherapy or in those with molecular persistence. The treatment of high-risk Acute Promyelocytic Leukaemia (APL) remains an area of contention as ATO is not approved for this indication. These patients have a greater risk of complications during remission induction with ATO including differentiation syndrome. The optimal approach is to incorporate chemotherapy early into the treatment schedule with either Gemtuzumab Ozogamicin (GO) as in the high-risk arm of the NCRI AML17 trial and MD Anderson Cancer Centre studies or Idarubicin as in the Australian APML4 study.

## Pre-ATO Era Studies in NCRI Trials

Following the MRC AML 12 trial, the combination of ATRA and chemotherapy became the standard induction treatment in newly diagnosed patients with APL in the UK (1). De-intensification of therapy became the priority for subsequent trials and the question of the optimal chemotherapy backbone to combine with ATRA was the subject of the NCRI AML15 trial. In that trial the standard MRC-based chemotherapy previously used in the AML12 trial comprising two courses of induction with daunorubicin, AraC and etoposide (ADE) with ATRA followed by consolidation with courses of MACE then MiDAC was randomized against the anthracycline and ATRA regimen (AIDA) developed by the PETHEMA and GIMEMA groups (2, 3). AIDA, which consisted of idarubicin and ATRA induction followed by 3 cycles of anthracycline-based consolidation followed by maintenance for 2 years, proved as effective and less myelosuppressive than MRC combination chemotherapy approach (CR rate 93% in both arms, 5 year overall survival of 84% vs 83%). The AIDA regimen proved less toxic with fewer deaths in remission, reduced supportive care requirements and reduced in-patient stay (4).

In the relapsed setting arsenic trioxide (ATO)had been successfully used as salvage therapy showing satisfactory outcomes in a number of studies (5–8) leading to its regulatory approval for relapsed APL in 2000 in the USA and a year later in Europe. In the AML15 trial, centralized sequential MRD monitoring with a sensitivity of up to 1:10^5^ to 1:10^6^ for good-quality samples had been introduced with samples taken after each course of chemotherapy. MRD was used to direct pre-emptive ATO therapy in patients with molecular persistence of disease or molecular relapse. This application of centralized MRD monitoring and pre-emptive therapy with ATO was associated with a significant reduction in the rate of hematological relapse when compared with the previous MRC AML12 trial (5% vs 12% at 3 years, *P* = .02) (9). The experience of ATO as salvage therapy paved the way for its evaluation as upfront therapy in the subsequent UK NCRI AML17 trial.

## AML17 Experience Using Attenuated ATO in Newly Diagnosed APL

An important exploratory study by the MD Anderson Cancer Center using ATRA-ATO with or without GO suggested that an essentially chemotherapy-free regime might be feasible for the upfront treatment of APL (10). The NCRI AML17 trial built on these findings to investigate the de-intensification of treatment by randomizing patients irrespective of their risk status between ATO-ATRA and the AIDA regimen. For high risk patients GO was also administered on day 1 in the ATO arm (11). The Italian and German collaboration (GIMEMA-AMLSG-SAL) performed the first randomized trial randomizing ATO-ATRA against the AIDA regimen (APL 0406 trial) and showed that ATO-ATRA resulted in superior overall survival (12, 13). Unlike the AML17 trial this study was confined to standard risk patients only excluding the 25% of patients with high risk disease

**Figure 1:**
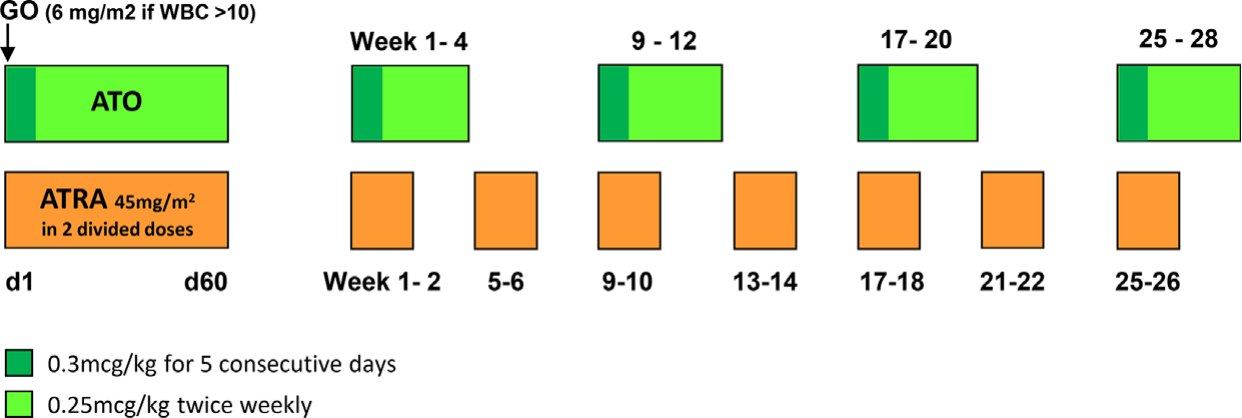
ATO plus ATRA dose schedule.

The ATO schedule used in AML17 was different to that used in the APL 0406 trial. The protocol was based upon previous experience of ATO in a multi-center European study of 104 patients with myelodysplastic syndrome and utilized a higher loading ATO dose in week 1 of induction and consolidation but less frequent dosing in subsequent weeks resulting in overall significantly lower ATO exposure and was well tolerated (11). As previously reported in detail (14) In AML17, ATRA was given in a daily divided oral dose of 45 mg/m^2^ until remission, or until day 60, and then in a 2 weeks on-2 weeks off schedule. ATO was given as a 1-h IV infusion using a loading dose of 0·3 mg/kg on days 1 to 5 of each induction and consolidation course, and then at a dose of 0·25 mg/kg twice weekly in weeks 2 to 8 of course 1 and weeks 2 to 4 of courses two to five (see **Figure 1**) (14). This contrasts with the daily ATO dosing schedule used in the APL 0406 trial, in the Australian APML4 study (15) and in the MD Anderson studies (10) which consist of ATO 0.15 mg/kg during induction until remission is achieved, typically 5 to 6 weeks. This is followed by four consolidation cycles, with a cycle length of 8 weeks with ATO being administered during weeks 1 to 4 at 0.15 mg/kg/d for 5 days per week. Thus the AML17 schedule offers a twice weekly schedule of ATO infusions during weeks 2 to 8 of induction and weeks 2 to 4 of the four courses of consolidation.

In AML17 high-risk patients also received a single dose of GO (6 mg/m^2^) on day 1 with the aim of preventing the leukocytosis occurring during ATO-based induction which increases the risk of differentiation syndrome. In addition 7 out of 86 standard risk patients in the ATO-ATRA arm received GO because of a rising white cell count. For patients randomized to AIDA, idarubicin was given intravenously at 12 mg/m^2^ on days 2, 4, 6, and 8 of course 1, and then at 5 mg/m^2^ on days 1 to 4 of course 2; mitoxantrone at 10 mg/m^2^ on days 1 to 4 of course 3, and idarubicin at 12 mg/m^2^ on day 1 of the final course. During induction treatment, ATO could be discontinued temporarily in the presence of differentiation syndrome, QT prolongation on ECG, or hepatotoxicity. Routine prophylaxis for differentiation syndrome was not recommended in the protocol, but prompt use of dexamethasone was suggested on clinical suspicion of differentiation syndrome.

MRD monitoring by reverse-transcription-quantitative PCR (RT-qPCR) was performed using bone marrow aspirates after each course of ATO and then 3 monthly for 3 years. The AIDA arm in AML17 had been de-intensified due to the omission of maintenance, the value of which had previously been questioned (16). Furthermore secondary AML had emerged as an important cause of treatment failure in the NCRI AML15 trial where 2 years of maintenance had been used (4).

As previously reported (14) from 05/2009 to 10/2013, 235 adult patients were randomized in the trial, the median age was 47 years (16–77 years); 57 had high risk APL of whom 30 received ATO and of these 28 received GO and 2 received idarubicin when GO was not available. 49 patients were over 60 years at diagnosis. Compliance with treatment was excellent at 99% in course 1 of ATO/ATRA and treatment was well tolerated with 60 day mortality of 5% overall and 1% in standard risk patients. During course 1 hyperbilirubinemia, and cardiac events were more common with ATRA and idarubicin, but there was no difference in raised liver enzyme levels between the two arms. Overall, liver toxicity appeared less frequent than in the GIMEMA-AMLSG-SAL protocol where grade 3–4 liver toxicity was reported in 63% of patients treated with ATRA-ATO (3). Differentiation syndrome was the most frequent complication seen and was reported in 25% of patients in the ATO-ATRA arm (23/86 standard risk and 7/30 high risk patients) which was not different to that reported in the AIDA arm. Cardiac events were more frequently reported with ATO-ATRA with grades 1 to 4 toxicity in 22% of patients and 3 of the 6 deaths by day 60 in that arm were cardiac events.

The results have been reported in detail by Burnett et al. (14). To summarize in terms of response, ATO-ATRA resulted in CR rate of 94% with no significant difference in the rate of complete remission (CR) between arms (ATO-ATRA 94%, AIDA 89%; HR 0.54 (0.21–1.34), p=0.18). Molecular complete remission (CR_MRD-_) was achieved in 91% of patients with ATO-ATRA and 88% with AIDA (p=0.43).No differences were seen in 5 year overall survival and was 92% (ATO-ATRA arm) vs 86% (AIDA arm) (HR 0.71; 95% CI, 0.33–1.50; p=0.4) (14). Of the patients who became MRD negative, molecular relapse free survival was significantly improved at 5 years being 97% with ATO-ATRA compared to 78% with AIDA (HR 0.25 (0.12–0.52) p=0.0002). No patient treated with ATO-ATRA who became molecularly negative actually relapsed whereas among AIDA treated patients the 5-year cumulative incidence of relapse was 20%. This resulted in a superior relapse free survival for ATO-ATRA (96% vs 86%, HR 0.43 (0.18–1.03) p=0.06). The reduction in relapse for ATRA-ATO was seen in both high and standard risk patients. Other advantages seen with ATO-ATRA included a reduced requirement for supportive care including less time spent in the hospital (33 versus 27 days in course 1). There were no reported cases of secondary AML reported in the ATO-ATRA arm of AML17 or indeed in the APL0406 trial (13, 14).

The lack of overall survival benefit for ATO-ATRA in AML17 contrasts with the outcomes reported in the GIMEMA-AMLSG-SAL APL0406trial (3, 13). One explanation for the lack of survival benefit in AML17 was that it was related to high compliance with centralized MRD monitoring resulting in the effective salvage of AIDA-treated patients which frequently took place at the time of molecular relapse. However, despite this lack of survival benefit the attenuated schedule of ATO did result in a similar response rate and RFS benefit as the conventional dose regimen but with lower drug acquisition costs and reduced burden of ATO administration which amounted to just 63 doses (14). These findings led to the approval of the attenuated AML17 schedule for re-imbursement by the National Institute for Clinical Excellence (NICE) in the UK. Also to its and its recommendation as an option for the upfront treatment of standard risk APL, in the ELN APL guidelines (17) and in the 2019 NCCN APL guidelines (18).

## Experience of Attenuated ATO Dosing in High-Risk and Relapsed APL

As previously discussed high risk APL patients were not included in the APL 0406 trial and ATO is not currently approved by the European Medicines Agency (EMA) for this group and is not reimbursed in the UK. It is generally agreed that cytotoxic chemotherapy should be administered with ATRA-ATO in high risk patients as the WBC may rapidly rise further after the initiation of ATRA with the risks of complications including differentiation syndrome. A number of approaches have been used. These include combining ATRA-ATO with GO as used in AML17 and by the MD Anderson Cancer Centre (10, 19) or with idarubicin as used in the Australian APML4 trial (15). The AML17 trial included a total of 57 high risk patients and their overall survival at 4 years was not significantly different from standard risk patients being 95% (95% CI 86%–98%) in standard risk compared with 87% (95% CI, 68%–95%) in high-risk patients Of the 28 high-risk patients in the ATO arm of AML17 who received the planned induction of ATRA, ATO and GO the 4 year survival was 89% (95% CI 70%–96%) (14). Unfortunately due to drug supply problems AML17 included only a relatively small sample size of high-risk patients and a prospective clinical trial (APOLLO trial) has been launched in Europe to verify whether the ATRA-ATO approach can be extended to the high-risk group. The NCRI AML17 result in high risk APL is however supported by a report from the MD Anderson Cancer Centre who also observed a low relapse risk in 187 standard- and high-risk (n=54) APL patients treated with ATO-ATRA, with the addition of GO (9 mg/m^2^)in the high risk group (19). The 5-year relapse-free and overall survival rates were 96%, and 88%, respectively. The Australian APML4 trial with idarubicin, ATRA and ATO treated 23 high-risk patients with a 5-year DFS of 95% and OS 87% (15).

The current approach to high-risk APL in the UK is with AIDA as ATO is not commissioned or reimbursed in this group. Recent guidance develop during the COVID pandemic has amended this recommendation to permit the use of ATO-ATRA for consolidation after receiving the first course of AIDA induction, the aim being to minimize neutropenia, hospital admission and the risk of nocosomial infection during subsequent courses of therapy. There were concerns that upfront ATO may be problematic for high-risk patients during the COVID pandemic because of the added risk of COVID-related pulmonary complications interacting with differentiation syndrome, moreover, the signs and symptoms of the two conditions show significant overlap which may create diagnostic uncertainty. However, the recent finding that dexamethasone is an effective treatment for COVID pneumonitis as well as its established efficacy in APL differentiation syndrome could simplify management decisions in this situation.

The attenuated ATO schedule has also been shown to be effective in patients relapsing after chemotherapy (20). Current recommendations for patients relapsing after initial treatment with chemotherapy are to re-induce with ATO-ATRA followed by consolidation with autologous transplantation in molecular CR if achieved (17). In AML17 a total of 189 patients were treated with AIDA, of whom 32 relapsed. These patients were treated with ATO-ATRA, receiving a median of 4 cycles (range, 1–5). Of the 31 patients treated, all achieved CR_MRD-_. Only 5 patients received additional chemotherapy therapy and that was after achieving molecular remission with ATO. Only 13 of 32 patients were transplanted in second remission (10 autograft, 3 allograft), including 4 of the 5 patients with CNS disease present at relapse. Of the 18 patients treated with ATO-ATRA who were not transplanted and did not receive any additional chemotherapy the 5-year overall survival was 88%. A similar approach of treating relapse after chemotherapy with prolonged ATO-ATRA therapy was reported by Cicconi et al. (21). They observed 5 relapses out of 20 patients who had achieved a complete molecular response to salvage ATO-ATRA. These results suggest that treatment of relapse with prolonged ATO-ATRA is an option for patients achieving molecular remission with ATO -ATRA who did not have CNS disease at relapse especially in patients who have had a long first remission (21).

## MRD Monitoring in APL in ATO Era

Molecular monitoring still plays an important role in patients treated with upfront ATO-ATRA however clinicians should be aware that its role, and consequently the recommended monitoring schedule, is different to that in patients treated with ATRA-CHT.

Most, but not all, patients treated with either ATO-ATRA or ATO-CHT achieve CR_MRD-_. In AML17, MRD-positivity at the end of treatment was seen in 3/106 (2.8%) patients who received at least one full course of ATO-ATRA and in 1/119 (1.3%) treated with ATRA-CHT (14). In contrast no patients in APL0406 had MRD-positivity after third consolidation (n=75 and 70 for ATRA-ATO and ATRA-CHT respectively) (13, 22). It is recommended that molecular monitoring should be performed until CR_MRD-_ is documented (17). This requires that both *PML-RARA* and reciprocal *RARA-PML* transcripts are undetected in two consecutive, technically adequate bone marrow aspirate samples.

The time to achievement of molecular negativity may differ between patients treated with ATO-ATRA and ATO-CHT. In AML17, the median time to CR_MRD-_ was 111 days with ATRA-ATO and 83 days with ATRA-CHT. The proportion of patients achieving molecular negativity by day 60 was also lower in patients treated with ATO-ATRA (56% vs 73%, p=0.03). The kinetics of molecular clearance did not differ between ATO-ATRA and ATO-CHT in APL0406, however fewer patients were evaluated in this analysis. It is not recommended to modify treatment for patients who have MRD-positivity after the first or second cycle of ATRA-ATO (17). For the small proportion of patients who have molecular persistence beyond the end of the third cycle, expert advice should be sought.

Finally, and perhaps most importantly, the rate of relapse is much lower with ATRA-ATO compared to ATRA-CHT as outlined above. In APL0406 only 2/75 (2.6%) patients in the ATO-ATRA arm relapsed and in AML17 there were no relapses in patients who had achieved CR_MRD-_. Therefore, sequential monitoring is not recommended for standard-risk patients who have been treated with frontline ATO-ATRA and who have achieved a documented CR_MRD-_ as the very low risk of relapse does not justify repeated bone marrow examinations (17). Patients with high-risk APL treated with ATO-based protocols should still receive molecular monitoring as the rate of relapse remains somewhat uncertain due to the relatively small number of patients treated in clinical trials to date. Patients treated with ATO-based salvage therapies following relapse after frontline ATRA-CHT treatment should continue to receive molecular monitoring as they remain at elevated risk of subsequent relapse, and pre-emptive treatment at molecular relapse may improve outcome (10, 14). For these patients, bone marrow monitoring every three months for at least two years after the completion of treatment is recommended (17).

## Conclusions

The attenuated ATO dosing schedule offers advantages of convenience for patients compared with conventional dosing regimen with evidence of reduced hepatotoxicity. Although no direct comparison has been made with the conventional dose regimen the attenuated schedule proved effective as first line therapy in both standard risk disease and also in high risk patients and following relapse after chemotherapy. The regimen offers advantage in that it reduces both the frequency of administration and the acquisition costs of ATO in UK.

## Data Availability Statement

The original contributions presented in the study are included in the article/supplementary material. Further inquiries can be directed to the corresponding author.

## Author Contributions

All authors listed have made a substantial, direct, and intellectual contribution to the work and approved it for publication.

## Funding

This work was supported by a Clinical Trial Award from Cancer Research UK and a Programme Grant for Applied Research from the National Institute of Health Research.

## Conflict of Interest

The authors declare that the research was conducted in the absence of any commercial or financial relationships that could be construed as a potential conflict of interest.

## References

[1] Burnett AK Grimwade D Solomon E Wheatley K Goldstone AH Presenting white blood cell count and kinetics of molecular remission predict prognosis in acute promyelocytic leukemia treated with all-trans retinoic acid: result of the Randomized MRC Trial. Blood (1999) 93(12):4131–43. 10.1182/blood.V93.12.4131.412k12_4131_4143 10361110

[2] Sanz MA Martín G Rayón C Esteve J González M Díaz-Mediavilla J A modified AIDA protocol with anthracycline-based consolidation results in high antileukemic efficacy and reduced toxicity in newly diagnosed PML/RARalpha-positive acute promyelocytic leukemia. PETHEMA group. Blood (1999) 94(9):3015–21. 10556184

[3] Lo-Coco F Avvisati G Vignetti M Breccia M Gallo E Rambaldi A Front-line treatment of acute promyelocytic leukemia with AIDA induction followed by risk-adapted consolidation for adults younger than 61 years: results of the AIDA-2000 trial of the GIMEMA Group. Blood (2010) 116(17):3171–9. 10.1182/blood-2010-03-276196 20644121

[4] Burnett AK Hills RK Grimwade D Jovanovic JV Craig J McMullin MF Inclusion of chemotherapy in addition to anthracycline in the treatment of acute promyelocytic leukaemia does not improve outcomes: results of the MRC AML15 trial. Leukemia (2013) 27(4):843–51. 10.1038/leu.2012.360 23222369

[5] Niu C Yan H Yu T Sun HP Liu JX Li X Studies on treatment of acute promyelocytic leukemia with arsenic trioxide: remission induction, follow-up, and molecular monitoring in 11 newly diagnosed and 47 relapsed acute promyelocytic leukemia patients. Blood (1999) 94(10):3315–24. 10.1182/blood.V94.10.3315.422k16_3315_3324 10552940

[6] Shen ZX Chen GQ Ni JH Li XS Xiong SM Qiu QY Use of arsenic trioxide (As2O3) in the treatment of acute promyelocytic leukemia (APL): II. Clinical efficacy and pharmacokinetics in relapsed patients. Blood (1997) 89(9):3354–60. 10.1182/blood.V89.9.3354 9129042

[7] Soignet SL Maslak P Wang ZG Jhanwar S Calleja E Dardashti L Complete remission after treatment of acute promyelocytic leukemia with arsenic trioxide. N Engl J Med (1998) 339(19):1341–8. 10.1056/NEJM199811053391901 9801394

[8] Soignet SL Frankel SR Douer D Tallman S Kantarjian H Calleja E United States multicenter study of arsenic trioxide in relapsed acute promyelocytic leukemia. J Clin Oncol (2001) 19(18):3852–60. 10.1200/JCO.2001.19.18.3852 11559723

[9] Grimwade D Jovanovic JV Hills RK Nugent EA Patel Y Flora R Prospective minimal residual disease monitoring to predict relapse of acute promyelocytic leukemia and to direct pre-emptive arsenic trioxide therapy. J Clin Oncol (2009) 27(22):3650–8. 10.1200/JCO.2008.20.1533 19506161

[10] Ravandi F Estey E Jones D Faderl S O’Brien S Fiorentino J Effective treatment of acute promyelocytic leukemia with all-trans-retinoic acid, arsenic trioxide, and gemtuzumab ozogamicin. J Clin Oncol (2009) 27(4):504–10. 10.1200/JCO.2008.18.6130 PMC4881307 19075265

[11] Vey N Dreyfus F Guerci M Fenaux P Dombret H Burnett A Trisenox® (arsenic trioxide) in Patients with Myelodysplastic Syndromes (MDS): Preliminary Results of a Phase I/II Study. Blood (2004) 104(11):1433. 10.1182/blood.V104.11.1433.1433

[12] Sanz MA Martín G González M León A Rayón C Rivas C Risk-adapted treatment of acute promyelocytic leukemia with all-trans-retinoic acid and anthracycline monochemotherapy: a multicenter study by the PETHEMA group. Blood (2004) 103(4):1237–43. 10.1182/blood-2003-07-2462 14576047

[13] Lo-Coco F Avvisati G Vignetti M Thiede C Orlando SM Iacobelli S Study Alliance Leukemia . Retinoic acid and arsenic trioxide for acute promyelocytic leukemia. New Engl J Med (2013) 369(2):111–21. 10.1056/NEJMoa1300874 23841729

[14] Burnett AK Russell NH Hills RK Bowen D Kell J Knapper S Arsenic trioxide and all-trans retinoic acid treatment for acute promyelocytic leukaemia in all risk groups (AML17): results of a randomised, controlled, phase 3 trial. Lancet Oncol (2015) 16(13):1295–305. 10.1016/S1470-2045(15)00193-X 26384238

[15] Iland HJ Bradstock K Supple SG Catalano A Collins M Hertzberg M All-trans-retinoic acid, idarubicin, and IV arsenic trioxide as initial therapy in acute promyelocytic leukemia (APML4). Blood (2012) 120(8):1570–752. 10.1182/blood-2012-02-410746 22715121

[16] Marzbani E Estey E Who benefits from maintenance therapy in acute promyelocytic leukemia? J Natl Compr Canc Netw (2012) 10(8):1023–8. 10.6004/jnccn.2012.0104 22878825

[17] Sanz MA Fenaux P Tallman MS Estey EH Löwenberg B Naoe T Management of acute promyelocytic leukemia: updated recommendations from an expert panel of the European LeukemiaNet. Blood (2019) 133(15):1630–43. 10.1182/blood-2019-01-894980 PMC6509567 30803991

[18] Tallman MS Wang ES Altman JK Appelbaum FR Bhatt VR Bixby D Acute Myeloid Leukemia, Version 3.2019, NCCN Clinical Practice Guidelines in Oncology. J Natl Compr Cancer Netw JNCCN (2019) 17(6):721–49. 10.6004/jnccn.2019.0028 31200351

[19] Abaza Y Kantarjian HM Garcia-Manero G Estey E Borthakur G Jabbour E Long-term outcome of acute promyelocytic leukemia treated with all-trans-retinoic acid, arsenic trioxide, and gemtuzumab. Blood (2017) 129:1275–83. 10.1182/blood-2016-09-736686 PMC5413297 28003274

[20] Russell N Burnett A Hills R Betteridge S Dennis M Jovanovic J Attenuated arsenic trioxide plus ATRA therapy for newly diagnosed and relapsed APL: long-term follow-up of the AML17 trial. Blood (2018) 132(13):1452–4. 10.1182/blood-2018-05-851824 PMC6225356 30097508

[21] Cicconi L Breccia M Franceschini L Latagliata R Molica M Divona M Prolonged treatment with arsenic trioxide (ATO) and all-trans-retinoic acid (ATRA) for relapsed acute promyelocytic leukemia previously treated with ATRA and chemotherapy. Ann Hematol (2018) 97(10):1797–802. 10.1007/s00277-018-3400-z 29951912

[22] Platzbecker U Avvisati G Cicconi L Thiede C Paoloni F Vignetti M Improved Outcomes With Retinoic Acid and Arsenic Trioxide Compared With Retinoic Acid and Chemotherapy in Non-High-Risk Acute Promyelocytic Leukemia: Final Results of the Randomized Italian-German APL0406 Trial. J Clin Oncol Off J Am Soc Clin Oncol (2017) 35(6):605–12. 10.1200/JCO.2016.67.1982 27400939

